# Functional and Structural Connectivity Correlates of Axial Symptom Outcomes After Pallidal Deep Brain Stimulation in Parkinson’s Disease

**DOI:** 10.3390/brainsci15111245

**Published:** 2025-11-20

**Authors:** Gilberto Perez Rodriguez Garcia, Erik Middlebrooks, Shanshan Mei, Takashi Tsuboi, Joshua Wong, Matthew Burns, Coralie de Hemptinne, Adolfo Ramirez-Zamora

**Affiliations:** 1 Norman Fixel Institute for Neurological Diseases, University of Florida, Gainesville, FL 32608, USA; gilberto.perezrodriguezgarcia@bmc.org (G.P.R.G.);; 2 Departments of Neuroradiology and Neurosurgery, Mayo Clinic, Jacksonville, FL 32224, USA; 3 Department of Neurology, Xuanwu Hospital, Capital Medical University, Beijing 100053, China; meishanshan@xwhosp.org; 4 Department of Neurology, Nagoya University, Nagoya 466-8550, Japan

**Keywords:** Parkinson’s disease, deep brain stimulation, globus pallidus interna, functional connectivity, structural connectivity, cerebellar crus II, axial symptoms, motor networks, connectomics

## Abstract

**Background/Objectives**: Deep brain stimulation (DBS) of the globus pallidus interna (GPi) is a safe and established therapy for management of refractory motor fluctuations and dyskinesia in Parkinson’s disease (PD). However, the relationship between stimulation site connectivity and improvement of axial gait symptoms remains poorly understood, particularly when stimulating in the GPi. This study investigated functional and structural connectivity patterns specifically associated with axial symptom outcomes following bilateral GPi-DBS, and, as a secondary exploratory analysis, examined whether Volumes of tissue activated (VTAs)-based connectivity related to overall UPDRS-III change. **Methods**: We retrospectively analyzed 19 PD patients who underwent bilateral GPi-DBS at the University of Florida (2002–2017). Unified Parkinson’s Disease Rating Scale (UPDRS-III) axial gait subscores were assessed at baseline and 36-month follow-up. VTAs were reconstructed using Lead-DBS and coregistered to Montreal Neurological Institute (MNI) space. Structural connectivity was evaluated with diffusion tractography, and functional connectivity was estimated using normative resting-state fMRI datasets. Correlations between VTA connectivity and clinical improvement were examined using Spearman correlation and voxelwise analyses. **Results**: Patients with axial improvement in motor scales demonstrated specific VTA connectivity to sensorimotor and supplementary motor networks, particularly lobule V and lobules I–IV of the cerebellum. These associations were specific to axial gait subscores. In contrast, worsening axial gait symptoms correlated with connectivity to cerebellar Crus II, cerebellum VIII, calcarine cortex, and thalamus (*p* < 0.05). Total UPDRS-III scores did not show a significant positive correlation with supplementary motor area or primary motor cortex connectivity; a non-significant trend was observed for VTA–M1 connectivity (R = 0.41, *p* = 0.078). Worsening total motor scores were associated with cerebellar Crus II and frontal–parietal networks. These findings suggest that distinct connectivity patterns underlie differential trajectories in axial and global motor outcomes following GPi-DBS. **Conclusions**: Distinct connectivity profiles might underlie axial gait symptom outcomes following GPi-DBS. Connectivity to motor and sensorimotor pathways supports improvement, whereas involvement of Crus II and occipital networks predicts worsening. Additional studies to confirm and expand on these findings are needed, but our results highlight the value of connectomic mapping for refining patient-specific targeting and developing future programming strategies.

## 1. Introduction

Deep brain stimulation (DBS) is a safe and effective treatment for management of motor fluctuations, complications (e.g., levodopa-induced dyskinesias, off-period dystonia, and unpredictable fluctuations in bradykinesia, rigidity, and tremor), and refractory tremor in Parkinson’s disease (PD). Improvement in motor symptoms and quality of life has been demonstrated in randomized controlled trials between the subthalamic nucleus (STN) and Globus Pallidus interna (GPi) [1]. The mechanism of action of DBS has been debated and expanded in recent years, and the therapeutic benefit of neurostimulation depends on its effects in regional anatomy and likely remote brain regions connected to the stimulation site [2,3,4]. These remote effects of DBS can be measured with both neurophysiological techniques [5,6] and neuroimaging [7,8]. Importantly, research indicates that specific areas of stimulation might correlate with unique clinical responses [9] and a better understanding of the differences in functional effects with certain stimulation parameters could potentially predict (and optimize) the clinical response [10].

Recent work has demonstrated that the connectivity profile of DBS targets correlates with clinical response in PD [11,12,13]. Stimulation within distinct subterritories of STN differentially modulates motor, gait, balance, and affective domains, reflecting functional segregation within the nucleus and its associated networks [14,15,16]. In GPi-DBS, pallidal stimulation reorganizes cortico–basal ganglia–thalamo–cortical circuitry, characterized by increased premotor cortical metabolism with suppression of pathological beta oscillations, reduced pallido–cortical coherence, and normalization of cortical phase–amplitude coupling [17,18,19]. Resting-state fMRI studies further show frequency-dependent alterations in GPi-seeded connectivity associated with motor improvement [20], while connectomic models can predict GPi-DBS outcomes and identify evoked responses as biomarkers of network engagement [12,21]. Although less extensively mapped than the STN, emerging sweet-spot and structural-connectivity analyses suggest functional heterogeneity within the GPi, with the sensorimotor region playing a dominant role in therapeutic efficacy [22]. In this study, we aimed to assess the associations between GPi Volumes of tissue activated (VTA)-based connectivity and axial gait motor symptoms (UPDRS-III items 27–30) at 36 months after bilateral GPi-DBS. A pre-specified secondary, exploratory analysis evaluated relationships between connectivity and overall UPDRS-III change.

## 2. Materials and Methods

### 2.1. Patient Selection

The study was approved by the University of Florida (UF) Institutional Review Board (IRB201901807, 16 July 2019). The study used the INFORM database which is a large movement disorders database at the University of Florida. We analyzed demographic, clinical, and surgical data of suitable subjects [23]. Patient records were anonymized and de-identified prior to analysis. Patients were eligible for enrollment if they had bilateral DBS placement at the UF Norman Fixel Institute for Neurological Disorders between 2002 to 2017 with complete data at long term follow-up. Target selection was based on multidisciplinary assessments [24]. Inclusion criteria were (1) bilateral placement of DBS, (2) fulfillment of the UK Parkinson’s disease Society Brain Bank Clinical diagnosis criteria [24,25], (3) patients with both on-med and off-med scores in preoperative assessment and postoperative 6-month (considered between 3 to 9 months) and 36-month (considered between 33 to 39 months) follow-up assessments. Exclusion criteria were (1) patients with more than two leads implanted on one side or with different stimulation targets between hemispheres; (2) patients who underwent two procedures on the same side (lead revision or replacement); and (3) patients with follow-up less than 30 months after the second (contralateral) implantation (defined as month 0). A cutoff year of 2017 was applied to ensure adequate long-term follow-up and to maintain consistency in hardware use by excluding newer systems such as the 330015 SenSight leads, thereby minimizing heterogeneity.

### 2.2. Surgical Procedure and Electrode Location

Preoperative imaging was used to determine the planned stereotactic coordinates of the GPi target before surgery for each specific patient. Nuclei were structurally identified by manually fitting a digitized and modified Schaltenbrand–Bailey atlas to subject’s MRI through the identification of white and grey matter [26]. Microelectrode recordings and monopolar testing during surgery led to adjustments of the functional target if necessary. All patients received Medtronic (Minneapolis, MN, USA) 3387 implants. The anatomical location of the DBS electrode was measured from a postoperative CT scan. The measured electrode position was reverse transformed into the normalized anterior commissure-posterior commissure (AC-PC) atlas space. Neurostimulators were placed approximately 4 weeks later and activated during the first clinical DBS programming visit. All patients underwent staged bilateral GPi-DBS implantation, with the second lead placed on average 13.4 ± 11.8 months after the first. The date of the second (contralateral) lead implantation was defined as month 0 for all longitudinal follow-up analyses, and clinical evaluation was performed at 36 months (allowable window 33–39 months) relative to the second surgery (see Table 1).

### 2.3. Assessments

The patients were assessed before surgery (baseline) and then at 36 months (36 M) after surgery. The baseline information included age, gender, age of onset, and age at DBS implantation. The clinical assessment included the Unified Parkinson’s disease Rating Scale (UPDRS) Part II and Part III. At baseline and during follow-up, we obtained motor data based on UPDRS-III scores in the off-medication conditions with on-DBS in long term follow-up assessments. The off-medication condition was defined as being off dopaminergic medications for 12 h. We defined an axial gait scores using UPDRS-III scores corresponding to the sum of the stand from chair score (item 27), posture score (item 28), Gait (item 29), and postural stability score (item 30) [27,28]. Daily dopaminergic treatment was defined as the L-dopa equivalent daily dose (LEDD), calculated using the method of Jost et al. [29]. Dopaminergic responsiveness was calculated with the following formula: (score (off-medication)—score (on-medication)) × 100/score (off-medication).

Group definition and thresholds. Patients were divided into two groups based on the direction of change in the axial UPDRS-III subscore at the 36-month assessment relative to baseline: an axial improvement group (decrease of ≥1 point) and an axial worsening group (increase of ≥1 point). Because the axial subscore (items 27–30) ranges from 0–16 with 1-point granularity per item, a 1-point change reflects a clinically visible shift on at least one axial item.

### 2.4. Imaging Technique and MRI Data Analysis

Lead-DBS software package version 2.3 (Lead-DBS GmbH, Berlin, Germany)  (http://www.lead-dbs.org, accessed on 1 October 2020) was utilized to localize DBS electrodes and model VTAs [30]. Pre-operative magnetization-prepared rapid gradient-echo (MP-RAGE) images and post-operative CT were co-registered using a two-stage linear registration using Advanced Normalization Tools v2.1 (University of Pennsylvania, Philadelphia, PA, USA; http://stnava.github.io/ANTs/, accessed on 1 October 2020) [31]. The MP-RAGE images were used as a basis for normalization into MNI_ICBM_2009b_NLIN_ASYM space [32] with the SyN registration method in Advanced Normalization Tools [31] using a five-stage nonlinear transform: two linear (rigid and affine) registrations, whole-brain nonlinear SyN-registration, and two nonlinear SyN-registrations with attention to the subcortical nuclei as defined by subcortical masks in Schonecker 2009 [32]. Identical transformation of the co-registered CT was then performed. The PaCER algorithm v1.0.7 (https://adhusch.github.io/PaCER, accessed on 1 October 2020) [33] was used for automatic electrode localization, followed by manual verification and refinement through visual inspection.

Utilizing each patient’s most effective programming settings, VTAs were generated, as described in Horn et al. [34]. The right-sided VTAs were nonlinearly mirrored to the left side. The VTAs for the two groups (worsening axial symptoms versus better axial symptoms) were then averaged and the center-of-gravity (COG) was then assessed for the resulting clusters using the “cluster” function of FSL v.6 (FMRIB Centre, University of Oxford, Oxford, UK, https://fsl.fmrib.ox.ac.uk/fsl/fslwiki/, accessed on 1 October 2020).

Diffusion tensor imaging was used to estimate structural connectivity from each patients’ VTA using a normative dataset of 32 healthy subjects in the Human Connectome Project imaged at Massachusetts General Hospital (https://ida.loni.usc.edu/, accessed on 1 October 2020). Using Lead-DBS software, fiber connectivity between the VTA and primary motor cortex (M1) and the supplementary motor area (SMA) was correlated with UPDRS change. Next, each VTA was used as a seed to generate fiber tracts for each patient using DSI Studio (http://dsi-studio.labsolver.org/, accessed on 1 October 2020). Tractography was based on a group average of 1021 subjects from the Human Connectome Project (2017 Q4, 1200-subject release). Tracking parameters included a qa threshold of 0.06 and angular threshold of 65 degrees with 1,000,000 seeds per VTA. The Automated Anatomical Labeling Atlas (AAL) [35] was used to assess the number of tracks terminating in each cortical and subcortical region. Number of tracks was then normalized for each patient by their total number of tracks generated. Regions-of-interest having <5% of total propagated paths in all patients were excluded from further analysis. Next, Spearman correlation was performed to assess correlation of fiber counts to each region-of-interest with axial score change using Prism v. 8.1.1 (GraphPad Software, San Diego, CA, USA). A *p* < 0.05 was considered statistically significant.

To assess functional connectivity correlates of axial symptom improvement, each left hemisphere and mirrored right hemisphere VTA was used as a seed to calculate functional connectivity. A total of 1000 healthy subjects in the Brain Genomics Superstruct Project (https://dataverse.harvard.edu/dataverse/GSP, accessed on 1 October 2020) were utilized using previously described methods [36]. Correlation between each VTA and all voxels was performed to generate r-maps that then underwent Fisher z-transformation. A two-group unpaired *t*-test was then performed using the individual connectivity maps to compare patients with worsening axial symptoms versus improved axial symptoms in Statistical Parametric Mapping (SPM) v12 (https://www.fil.ion.ucl.ac.uk/spm/, accessed on 1 October 2020). Resultant *t*-score maps were thresholded to correspond to a *p* < 0.05.

### 2.5. Statistical Analysis

Univariate descriptive analyses were used to report sample-level demographic and clinical characteristics. The independent samples two-tailed *t*-test for normal distribution data or Mann–Whitney U test for non-normal data were used to compare the age of onset, age at surgery, duration of follow-up, duration of PD and UPDRS-III score between groups. Chi-square test was used to compare gender between groups. Other differences between groups were analyzed by means of the Mann–Whitney test. Two-sided *p* values of less than 0.05 indicated statistical significance. To reduce Type I error, we used false discovery rate (FDR) correction across the connectomic analyses. Analyses were performed using the statistical software IBM SPSS, version 23.

## 3. Results

### 3.1. Study Population

Nineteen GPi-DBS patients with complete data were enrolled; two groups were identified based on the predefined axial gait subscore criteria at the 36-month follow-up (improvement group, n = 10; worsening group, n = 9; Table 1). All surgeries were staged, and follow-up was anchored to the second (contralateral) surgery (month 0). The 36-month assessment occurred a mean (±SD) of 35.4 ± 3.3 months after the second surgery (window 33–39 months). The main demographic and clinical characteristics are summarized in Table 1. No significant baseline differences were observed between groups.

### 3.2. Contact Position

**Figure 1:**
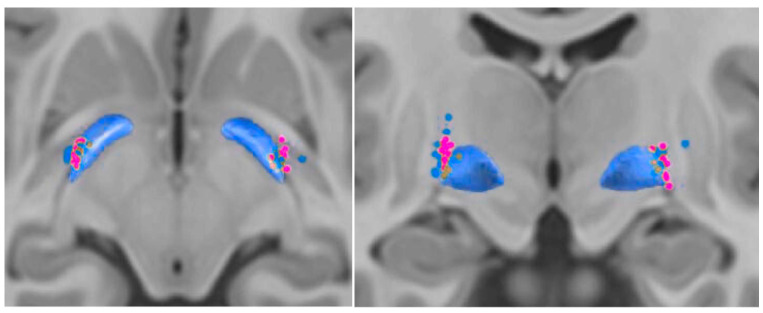
Active contact positions and volume of tissue activated (VTA) in patients with better vs. worsening axial symptoms. Active stimulation contacts are shown for the worsening group (pink) and the better outcome group (blue). VTAs associated with worsening axial symptoms were located slightly more anterior and medial on average.

The Montreal Neurological Institute (MNI) coordinates for the center of gravity (COG) of the VTAs in the worsening group were located slightly more anterior and medial (worsening COG: −22.6/−8.2/−2.3) compared to those in the improvement group (−22.9/−8.8/−2.3; Figure 1).

### 3.3. Structural Connectivity and Fiber Tract Count

In our cohort, as a secondary exploratory analysis, we examined whether VTA-based connectivity to motor cortical regions was related to overall UPDRS-III change (ΔUPDRS).

#### 3.3.1. Axial Gait Scores

**Figure 2:**
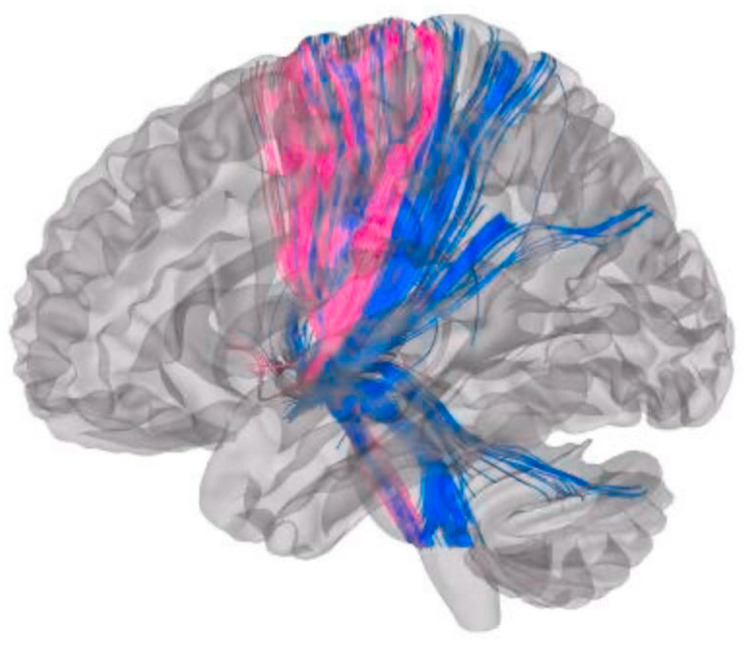
Tractography in two representative patients with divergent axial outcomes. Whole-brain tractography is shown for Subject 3 (pink) with an axial score improvement of 2 points, demonstrating minimal cerebellar connectivity, and for Subject 9 (blue) with an axial score worsening of 10 points, demonstrating prominent cerebellar fiber projections. These cases illustrate the variability of cerebellar involvement in relation to axial symptom progression.

Worsening axial symptoms were most strongly correlated (Spearman correlations of Axial Score Change OFF Med) with fiber tract connectivity to the cerebellum Crus II (Spearman r = 0.61, *p* = 0.006), followed by cerebellum lobule VIII (r = 0.52, *p* = 0.02), calcarine cortex (r = 0.59, *p* = 0.007), and thalamus (r = 0.48, *p* = 0.04). Group-level comparisons further supported these findings (Table 2), showing that patients with worse axial outcomes exhibited significantly greater connectivity to the ipsilateral cerebellum Crus II (47.6 vs. 31.6, *p* = 0.02), whereas those with better outcomes demonstrated higher connectivity to the ipsilateral putamen (68.6 vs. 46.7, *p* = 0.001) and pallidum (116.2 vs. 96.9, *p* = 0.005). In contrast, improvement in axial score was associated with cerebellar lobule V and, to a lesser extent, lobules I–IV and lobule VI, whereas worsening was associated with the nodulus. Improvement in axial score also correlated with connectivity to the sensorimotor and supplementary motor networks. Representative tractography further highlights this association, with Subject 3 (pink) showing axial improvement and no cerebellar connectivity, whereas Subject 9 (blue) demonstrated worsening axial symptoms with prominent cerebellar fiber projections (Figure 2).

#### 3.3.2. UPDRS Improvement

**Figure 3:**
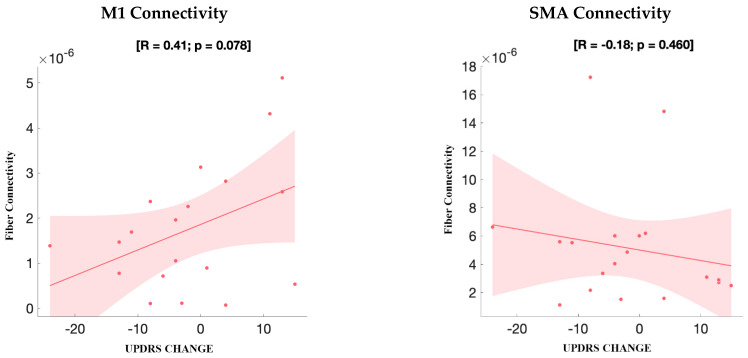
Secondary analysis: correlation between fiber connectivity and UPDRS-III change. Scatterplots show the relationship between motor improvement (ΔUPDRS) and structural fiber connectivity from GPi VTA seeds to motor regions. A positive correlation was observed between ΔUPDRS improvement and M1 connectivity (left; R = 0.41, *p* = 0.078), although this did not reach statistical significance. No significant correlation was found between SMA connectivity and ΔUPDRS improvement (right; R = −0.18, *p* = 0.460).

Improvement in axial gait subscores was associated with connectivity to sensorimotor and supplementary motor regions; however, when considering total UPDRS-III scores, no significant positive correlation was observed with connectivity to the SMA or M1. VTA–M1 connectivity showed a positive trend (R = 0.41, *p* = 0.078) that did not reach statistical significance, while VTA–SMA connectivity demonstrated no significant association (R = −0.18, *p* = 0.460). Connectivity to other cortical and subcortical regions showed no significant association with total UPDRS-III change (Figure 3). A trend toward altered connectivity involving cerebellar Crus II was observed in association with UPDRS change (*p* > 0.05). This region’s potential role in axial symptom modulation warrants further investigation.

### 3.4. Functional Connectivity

Functional MRI–based analyses were then performed to evaluate group differences in functional connectivity patterns between patients with better versus worse axial outcomes.

Worsening UPDRS scores correspond to more extensive cerebellar connectivity relative to regions associated with axial score improvement; overlapping areas are noted within lobules V and VI, with additional worsening-related regions including lobules I–IV, Crus I, and lobule IX.

**Figure 4:**
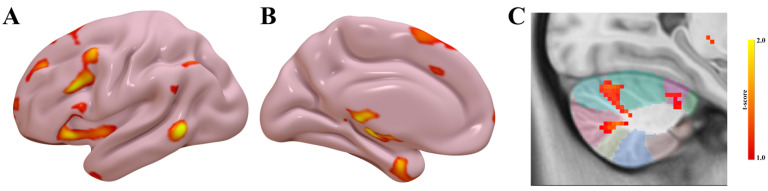
fMRI connectivity differences in patients with worsening axial symptoms. Group comparison of functional connectivity demonstrates greater connectivity in the worsening axial symptom group relative to the better axial symptom group. Lateral (**A**) and medial (**B**) hemisphere projections highlight increased connectivity in multiple cortical regions, including the supplementary motor area, middle frontal gyrus, and superior frontal gyrus. Increased connectivity was also observed in cerebellar regions (**C**), including lobules IV, VI, and Crus I–II. The cerebellar SUIT atlas is overlaid for anatomical reference.

Group comparisons of functional connectivity (Figure 4) further demonstrated greater connectivity in the worsening axial symptom group involving the supplementary motor area, middle frontal gyrus, and superior frontal gyrus. Enhanced cerebellar connectivity was also observed, particularly within lobules IV, VI, and Crus I–II.

## 4. Discussion

STN and GPi are the most common targets for neuromodulation therapy for refractory symptoms and motor complications in PD. The STN surgical target allows for a larger reduction in dopaminergic medication compared to GPi DBS with similar effects on motor disability and quality of life [37]. While both targets reduce dyskinesia in PD patients, the dyskinesia reduction associated with STN DBS is typically attributed to decreased medication dose [38]. Recent studies have begun to examine the functional connectivity patterns relating to DBS lead placement and corresponding stimulation of various surrounding brain regions to predict clinical outcomes. STN-DBS has been shown to directly activate the supplementary motor area, with evidence indicating that modulation of a complex basal ganglia network of spatially distributed motor regions contributes to its therapeutic effects on motor symptoms 38 [18,39,40]. The spatial activation patterns resulting from GPi DBS have yet to be researched to the same extent as STN DBS [20,41,42,43].

In this retrospective study, we found that fiber tract connectivity to the Cerebellum Crus 2 is most strongly correlated with worsened axial symptoms, whereas connectivity to the sensorimotor network and SMA in lobule V and partially in lobules I-IV is associated with improvement in axial scores. Crus II is part of the posterolateral cerebellum, which is functionally involved in cognitive and visuospatial domains [44,45,46]. Its hyperconnectivity in patients with worsening outcomes may reflect maladaptive recruitment of nonmotor cerebellar regions during gait and postural tasks [47,48]. In contrast, cerebellar lobules I–IV and V are sensorimotor in function, and their engagement likely contributes to improved axial motor control following GPi stimulation [49,50]. Furthermore, the group showing worsened axial symptoms exhibited VTA located more anterior and slightly more medial than the group showing improved axial symptoms. Regarding changes in UPDRS scores, more extensive cerebellar connectivity and connectivity to frontal, SMA, and inferior parietal lobule were associated with worse scores. Interestingly, the data showed some overlap between improved axial scores and worsened UPDRS scores. The observed correlation between GPi-DBS and improved axial scores via sensorimotor and SMA connectivity may reflect differences in network modulation compared to STN-DBS. GPi stimulation may exert more direct influence on axial pathways through basal ganglia-cerebellar and pallido-cortical loops, whereas STN-DBS is more influenced by medication reduction and may insufficiently engage axial control networks [20,51,52].

Connectivity measures have gained recent attention due to the clinical and predictive capabilities of mapping the spatial networks connected to DBS stimulation sites. The study of connectomics focuses on the structural and functional connectivity of brain regions to ultimately create a fully mapped human connectome [53]. Horn et al. utilized data from two such connectivity measures, diffusion tractography and functional connectivity, to compile a “connectivity profile” of STN DBS in PD patients in order to predict clinical outcomes [11]. The study found a positive correlation between the extent to which the PD patient’s DBS connectivity profile resembled the map of VTAs and electrode placement constructed from a larger dataset and the patients’ clinical outcomes. Diffusion tractography has been used primarily as a non-invasive method for examining white matter pathways and networks of brain regions activated in PD patients [53]. The significance of identifying functional and structural connectivity patterns in various brain regions lies in the clinical applications of predicting DBS outcomes based on spatial lead placement locations. Both deterministic and probabilistic methods of tractography identified corticopallidal and corticosubthalamic pathways across GPi and STN target regions [54]. The ipsilateral GPi and posterosuperolateral aspect of ipsilateral STN showed networking with the sensorimotor cortex. However, Muller et al. suggests the superiority of probabilistic tractography over deterministic tractography due to its increased sensitivity and ability to detect larger quantity of fibers in their study delineating the pallidal sensorimotor region in PD patients [54].

### 4.1. Integration of Stimulation Parameters and Connectivity Insights

Prior studies indicate that incorporating stimulation parameters, specifically modeling the VTA derived from electrode coordinates and chronic stimulation settings, provides superior predictive power compared to anatomical coordinates alone [11,55]. Our findings align with this framework, as VTA-derived connectivity profiles exhibited distinct patterns between patients with axial gait improvement and those with worsening symptoms, despite comparable baseline clinical characteristics and electrode placements.

Connectivity-based approaches also refine understanding of outcome variability by identifying network-level correlates of clinical benefit. Favorable results have been consistently associated with engagement of hyperdirect projections linking basal ganglia to primary motor and premotor cortices, whereas connections to associative or limbic regions have been linked to neuropsychiatric sequelae [11,56]. In our cohort, sensorimotor and SMA connectivity predicted axial improvement, supporting the therapeutic relevance of motor cortical influence on GPi activity, potentially mediated through cortico–subthalamo–pallidal or direct cortico–pallidal projections [57,58,59]. Conversely, cerebellar Crus II and calcarine involvement in worsening outcomes may reflect maladaptive visuomotor or cerebellar contributions to gait and postural control.

Notably, the calcarine cortex, a primary visual region, has seldom been implicated in DBS outcomes. However, metabolic imaging studies in multiple sclerosis demonstrate that calcarine glucose uptake correlates with walking speed, suggesting visual–motor integration influences locomotor performance [60]. Our results similarly raise the possibility that occipital–visual networks may modulate axial gait symptoms in PD and could be inadvertently engaged during GPi stimulation. This observation warrants further investigation using patient-specific imaging and task-based paradigms to clarify the role of visuomotor circuits in axial symptom modulation. Additionally, while we focused on fiber count to assess structural connectivity density, future studies incorporating FA and MD may yield further insight into the integrity of DBS-relevant tracts.

### 4.2. Strengths and Limitations

A key strength of this study is its innovative integration of anatomical lead localization with normative structural and functional connectivity analyses, allowing comprehensive mapping of GPi DBS effects on axial symptoms beyond focal electrode placement. The long-term follow-up at 36 months and standardized assessment using UPDRS-III and axial gait subscores provide clinically meaningful outcome data. The use of advanced imaging pipelines (Lead-DBS, PaCER) and normative connectomic resources enhances reproducibility and aligns the methodology with emerging standards in network-based DBS research, underscoring the relevance of these findings to future targeting and programming strategies.

Limitations include the retrospective design and small sample size, which may limit the ability to detect more subtle associations or perform extensive subgroup analyses. We cannot discount the challenges related to heterogeneity in disease progression in our cohort. The use of normative rather than patient-specific connectivity data may not capture individual disease-related network variability; however, normative datasets remain widely used and facilitate cross-cohort comparability. Additionally, modeled VTAs were based on chronic stimulation settings and may not account for dynamic programming adjustments over time. While the minimal clinically important difference (MCID) for UPDRS-III (±4.5 points) provides an important clinical benchmark, we opted to dichotomize patients based on the directionality of change (improvement vs. worsening) rather than apply a strict MCID threshold. This approach was chosen to reflect the full spectrum of postoperative clinical trajectories, including subtle but directionally meaningful changes, and to avoid potential exclusion of patients whose functional shifts may still represent biologically relevant phenomena. Given the exploratory nature of our analysis and the modest sample size, this strategy allowed for greater inclusivity and interpretability of network-based correlations. Nevertheless, future studies with larger cohorts may benefit from incorporating MCID-based dichotomization to refine responder classification and validate these findings. Despite these factors, the study provides valuable insights into GPi connectivity correlates of axial gait symptom outcomes and establishes a framework for future prospective studies.

## 5. Conclusions

This study demonstrates that GPi DBS outcomes for axial gait symptoms in PD are associated with distinct connectivity patterns, particularly involving cerebellar Crus II, lobules I–VI, and sensorimotor networks. Axial improvement correlated with connectivity to sensorimotor and supplementary motor regions, whereas worsened outcomes were linked to anterior-medial VTAs and stronger connectivity to Crus II and occipital networks, including the calcarine cortex. These findings highlight the potential value of incorporating stimulation parameters and connectomic mapping into predictive models for patient-specific targeting and postoperative programming. Future prospective studies using patient-specific tractography and multimodal imaging are warranted to validate these associations and refine connectivity-guided approaches to GPi DBS for axial motor dysfunction in PD.

## Figures and Tables

**Table 1:** Main baseline clinical characteristics of the patients with PD involved in the long-term study *.

	Improving (n = 10) Mean ± SD (Range)	Worsening (n = 9) Mean ± SD (Range)	*p*-Value
**Gender, M/F**	7/3	6/3	1.000
**Age of onset (years)**	45.0 ± 6.6 (37–55)	47.9 ± 7.5 (37–59)	0.384
**Age at surgery (years)**	58.7 ± 8.5 (47–68)	62.4 ± 6.4 (55–74)	0.297
**Follow-up from baseline to the** **36-month timepoint (months)**	53.2 ± 12.9 (43.1– 85.6)	55.0 ± 10.4 (45.2–73.5)	0.740
**Duration of PD at baseline (years)**	21.0 ± 5.3 (13–21)	24.4 ± 7.9 (12–34)	0.274
UPDRS-III baseline **			
**Off-medication**	43.6 ±11.2 (28–68)	41.9 ± 6.6 (34–54)	0.695
**On-medication**	26.2 ±13.2 (15–58)	23.4 ± 10.7 (8–42)	0.627
**Axial gait score**			
**Off-medication**	6.8 ± 2.2 (4–10)	5.1 ± 3.1 (0–10)	0.185
**On-medication**	3.8 ± 3.0 (0–9)	3.2 ± 2.7 (0–7)	0.666
**Hoehn & Yahr**			
**Off-medication**	3.0 ± 0.7 (2–4)	2.8 ± 0.6 (2–4)	0.702
**On-medication**	2.6 ± 0.6 (2–4)	2.5 ± 0.6 (2–4)	0.737
**LEDD (mg)**	1299.6 ± 655.9 (300–2375)	1169.8 ± 698.4 (532–2429)	0.681

* Plus-minus values are means ± SD. Baseline variables were compared between the two groups with the use of a two-group *t*-test, Mann–Whitney U test, and chi-square test. ** Scores on the Unified Parkinson’s Disease Rating Scale III (UPDRS-III) range from 0 to 108, with higher scores indicating more severe disease.

**Table 2:** Comparison of fiber tract count between worse patient group and better patient (unpaired *t*-test; only statistically significant areas shown).

ROI	Mean (Better)	Mean (Worse)	Δ (Better–Worse)	t (df = 17)	*p*	Pooled SD	95% CI for Δ	Hedges’ g
Cerebellum Crus 2 (Ipsilateral)	31.6	47.6	−16.0	2.53	0.02	13.8	−29.3 to −2.7	−1.11
Putamen (Ipsilateral)	68.6	46.7	+21.9	3.92	0.001	12.2	+10.1 to +33.7	+1.72
Pallidum (Ipsilateral)	116.2	96.9	+19.3	3.00	0.005	14.0	+5.7 to +32.9	+1.32

Note. Values are reconstructed from reported *p* values using equal-variance two-sample *t* tests; estimates should be interpreted as approximate.

## Data Availability

The data presented in this study are available on reasonable request from the corresponding author. Imaging datasets were derived from normative public resources (Human Connectome Project, Brain Genomics Superstruct Project).

## Abbreviations

The following abbreviations are used in this manuscript:

AC–PC	Anterior commissure–posterior commissure
AAL	Automated Anatomical Labeling (atlas)
ANTs	Advanced Normalization Tools
BOLD	Blood oxygen level–dependent
COG	Center of gravity
DBS	Deep brain stimulation
DSI	Diffusion Spectrum Imaging
fMRI	Functional magnetic resonance imaging
FSL	FMRIB Software Library
GPi	Globus pallidus internus
GSP	Brain Genomics Superstruct Project
HCP	Human Connectome Project
INFORM	Movement disorders database at the University of Florida
LEDD	Levodopa equivalent daily dose
LFP	Local field potential
M1	Primary motor cortex
MEG	Magnetoencephalography
MNI	Montreal Neurological Institute (template space)
MP-RAGE	Magnetization-prepared rapid gradient-echo
MRI	Magnetic resonance imaging
PaCER	Automated method for electrode trajectory and contact reconstruction
PD	Parkinson’s disease
QA	Quantitative anisotropy
ROI	Region of interest
SD	Standard deviation
SMA	Supplementary motor area
SPM	Statistical Parametric Mapping
STN	Subthalamic nucleus
SUIT	Spatially Unbiased Infratentorial Template (cerebellar atlas)
UPDRS	Unified Parkinson’s Disease Rating Scale
VTA	Volume(s) of tissue activated
